# *S*-palmitoylation of NOD2 controls its localization to the plasma membrane

**DOI:** 10.1016/j.jlr.2021.100097

**Published:** 2021-07-20

**Authors:** Charneal L. Dixon, Gregory D. Fairn

**Affiliations:** 1 Keenan Research Centre for Biomedical Science, St. Michael’s Hospital, Unity Health Toronto, Toronto, Ontario, Canada; 2 Department of Biochemistry, University of Toronto, Toronto, Ontario, Canada; 3 Department of Pathology, Dalhousie University, Halifax, Ontario, Canada

**Figure:**
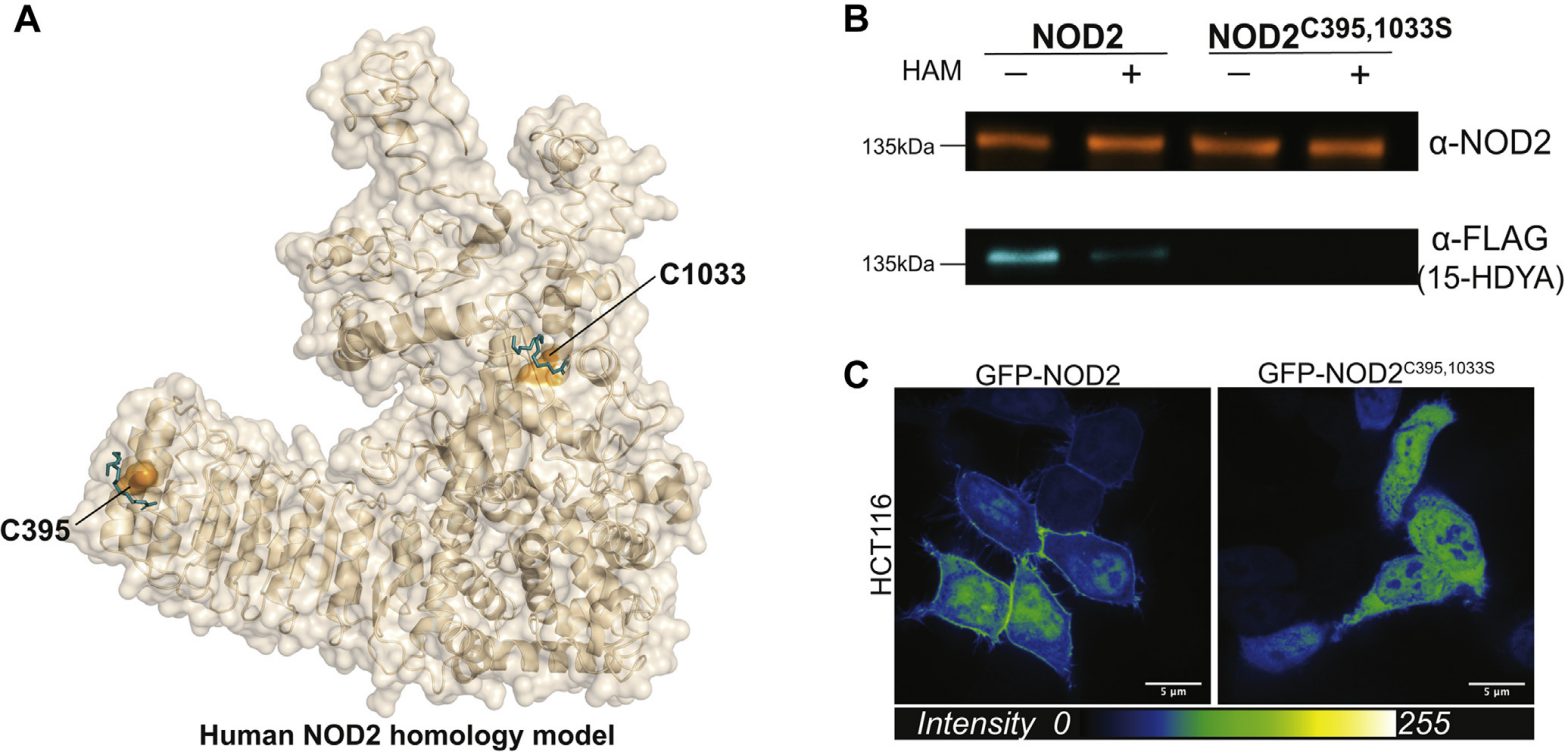

Nucleotide-binding and oligomerization domain containing protein 2 (NOD2) is a cytosolic pattern-recognition receptor that detects intracellular peptidoglycan (muramyl dipeptide) from bacteria. Membrane association of NOD2 is essential for its ability to activate nuclear factor κB and mitogen-activated protein kinase signaling pathways via the kinase, receptor-interacting serine/threonine-protein kinase 2. The post-translational addition of palmitate to NOD2 results in an acylated protein with increased affinity for membrane bilayers (1). Palmitoylation of cysteine residues (shown in orange) at positions C395 and C1033 *en face* of the structural model (A) based on the crystal structure of rabbit NOD2 (PDB ID: 5IRL) (2) is mediated by the protein acyltransferase enzyme zDHHC5 (1). The model suggests that this face of the protein is juxtaposed to the plasmalemmal surface. Palmitoylation of the expressed GFP-NOD2^WT^ and GFP-NOD2^C395,1033S^ in HCT116 cells was further characterized using a biorthogonal chemical reporter assay (B) (3). Here, 15-hexadecynoic acid (15-HDYA), also referred to as alkynyl palmitic acid, was metabolically incorporated into cells. The 15-HDYA covalently attached to the immunocaptured GFP-NOD2 proteins was reacted with azide-PEG_3_-FLAG via a copper-catalyzed azide–alkyne cycloaddition reaction. The proteins were resolved by SDS-PAGE and subsequently detected by an anti-FLAG antibody. The metabolic label, which was incorporated into NOD2^WT^ and was notably absent in the double cysteine mutant, was removed by treatment with 2.5% hydroxylamine that hydrolyzes the 15-DYA–protein thioester linkage. Finally, the palmitoylation-deficient GFP-NOD2^C395,1033S^ does not localize to the plasma membrane in HCT116 cells (C). Mutations that disrupt S-palmitoylation are associated with severe immunologic and inflammatory diseases such as Crohn’s disease, whereas mutations associated with Blau syndrome demonstrate increased S-palmitoylation and membrane localization (1).

**EQUIPMENT:** The following equipment were used: A Zeiss Axiovert 200M microscope with a Hamamatsu ImageEM x2 camera, Yokogawa spinning disc, and a 63× 1.4 NA oil objective.

**REAGENTS:** Alkynyl-palmitate (Click Chemistry Tools), azide-PEG_3_-FLAG (Jena Bioscience), hydroxylamine, and other copper-catalyzed azide–alkyne cycloaddition reagents (TCEP, CuSO_4_, and TBTA) were obtained from MilliporeSigma.

**SOFTWARE:** The following software were used: MetaMorph, ImageJ, Python, PyMOL, and AutoDock (4).
